# Effect of nanopatterning on mechanical properties of Lithium anode

**DOI:** 10.1038/s41598-018-20773-8

**Published:** 2018-02-06

**Authors:** Colin Campbell, Yong Min Lee, Kuk Young Cho, Young-Gi Lee, Byeongdu Lee, Charudatta Phatak, Seungbum Hong

**Affiliations:** 1 Materials Science Division, Argonne National Laboratory, Lemont, IL 60439 USA; 2 Department of Materials Science and Engineering, Northwestern University, Evanston, IL 60208 USA; 3 Department of Energy Science and Engineering, Daegu Gyeongbuk Institute of Science and Technology (DGIST), Daegu, 42988 Korea; 4 Deparment of Materials Science and Chemical Engineering, Hanyang University, Ansan, 15588 Korea; 5 ICT Materials and Components Research Laboratory, ETRI, Korea; 6 X-ray Science Division, Argonne National Laboratory, Lemont, IL 60439 USA; 7 Department of Materials Science and Engineering, KAIST, Daejeon, 34141 Korea

## Abstract

One of the challenges in developing Lithium anodes for Lithium ion batteries (LIB) is controlling the formation of Li dendrites during cycling of the battery. Nanostructuring and nanopatterning of electrodes shows a promising way to suppress the growth of Li dendrites. However, in order to control this behavior, a fundamental understanding of the effect of nanopatterning on the electro-mechanical properties of Li metal is necessary. In this paper, we have investigated the mechanical and wear properties of Li metal using Atomic Force Microscopy (AFM) in an airtight cell. By using different load regimes, we determined the mechanical properties of Li metal. We show that as a result of nanopatterning, Li metal surface underwent work hardening due to residual compressive stress. The presence of such stresses can help to improve cycle lifetime of LIBs with Li anodes and obtain very high energy densities. *Keywords:* Nanopatterning, mechanical properties, Li anode, Lithium ion batteries

## Introduction

Development of high-performance energy storage solution for applications ranging from portable electronics, and emission-free electric vehicles to emerging smart-grids is one of the important challenges facing modern economy^1,2^. Amongst other technologies, those based on Lithium-ion batteries (LIBs) have been most successful in meeting the energy density and power output requirements. However, due to an ever- increasing demand for higher energy density and smaller, portable packaging limits of even LIBs are being reached. Technologies such as inexpensive electric cars with comparable drive distances to current internal combustion engine cars still remain out of reach with our current energy storage capabilities^3,4^. This has been partly due to materials issues in LIBs that result in limited charge/discharge rate, stability, safety and operating temperature range. Although advancements in all areas of LIBs are of interest, the research into anode materials has remained relatively static: currently carbon-based materials, especially graphite, are the some of the most common anodes, with graphite at a theoretical specific capacity of 372 mAh/g^5^. Lithium metal, for comparison, has more than ten times the theoretical specific capacity of graphite, and a lower reduction potential^6^. These features provide for a greater energy storage per unit mass when compared with cells constructed using the more common graphite anode. This can result in a longer battery lifetime in existing electronics and higher-energy or even higher-power applications could be realized.

The primary obstacle in using Li anodes is the uncontrolled growth of Li dendrites, which occurs after repeated charging/discharging of the battery^7,8^. Li dendrite growth can lead to faded performance of the battery causing accelerated diffusion losses and ultimately catastrophic failure by short-circuiting of the battery. While Li metal rechargeable batteries have not been produced commercially due to safety and longevity concerns, many advances that inhibit the formation of dendrites have been developed. Recently, it has been demonstrated that mechanically modifying the surface of a lithium anode improves its safe cycle lifetime^9^. While the physical mechanisms of this cycle life increase are not fully understood, it is possible that other modified surface geometries provide similarly improved lifetimes, so it is of interest to know the mechanical properties of lithium metal because of their effect on forming these surface features, particularly if modifications can be made to take advantage of this. One advantage to this method is that in many cases it can be combined with other methods of improving stability, such as changing electrolyte composition to form a less dendrite-prone Solid Electrolyte Interphase (SEI)^10,11^, or using an additional surface layer above the anode^12,13^. Similar work has been reported on nanostructuring of cathode materials for enhanced performance of LIBs^14–16^. Generating small-scale particles of lithium iron phosphate (Li_x_FePO_4_) allowed for higher-power applications by decreasing the diffusion length involved in the charge/discharge of the cell^17^. Instead of enhancing the power supplied at a given current when compared with an unprocessed electrode, we hope to use nanopatterning to effectively slow the kinetics responsible for runaway dendritic failure^18^. Patterning electrodes is something that could theoretically be introduced into a battery assembly process at little expense (that of a chemically compatible stamp, for example), while simultaneously improving battery lifetime and/or the safe, repeatable limits of power supply.

In the work by Park *et al*., a template (micro-needle roller) was used to indent a sample of lithium metal to produce a micropatterned anode^9^. As a result, diminished Li dendrite growth was observed during cell operation, which was well supported by big current density differences between the Li metal surface and within the patterns with the help of simulation results. There must be some positive effects of increased lithium electrode area that reduces the effective Li plating current densities. However, simple enlargement of electrode area such as using lithium metal powder showed very limited enhancement of cycle performance^19^. Thus, the shapes, dimensions, and numbers of patterns on the lithium metal should deliberately be investigated depending on applications. The large-area small-scale indentation of Li metal is analogous to shot-peening methods in metallurgy, where material is lightly plastically deformed by repeated impacts to introduce a degree of residual compressive stress into the surface layer of the material^20^. This residual compressive stress also has relevance to the formation of dendrites in lithium metal. Prior theoretical work has established an effective mechanical overpotential based on the stresses at the surface and surface energy in the case of a conformal surface layer^21^. This was then applied to a periodically distorted interface of an anode and conformal coating, finding that a coating with a shear modulus greater than or equal to twice that of lithium was sufficient to suppress dendrite growth by promoting deposition in the “valley” and suppressing it at the tip^21^. Although the 2D calculation precludes a numerically exact answer, calculations in related work that corrects the previous model to ensure a neutral interface suggests that in a state free of residual tensile stress, external compressive stress (as exerted by newly deposited Li at the interface, for example) is suggested to eliminate dendrite formation entirely by redistributing current (in the assumed limit of homogenous electrolyte concentration)^22^. Nevertheless, this finding is significant: stress on the order of 500 MPa exerted at the tip was found to reduce the current at the tip to less than 1% that deposited in the valleys around a protrusion. This phenomenon is also demonstrated experimentally, as shot peening has been shown previously to increase the long-term corrosion resistance of materials which suggests that these surface modifications can result in a slower electrochemical reaction^23,24^. Futhermore, these results indicate a relatively long-term enhancement of corrosion resistance, suggesting a cause that is maintained for some depth beneath the original surface, which is more consistent with residual compressive stress than it is with the topological modification caused by shot-peening^23,24^. By utilizing such surface modification methods to reduce the electrochemical activity of a surface, the number of high-activity sites at the surface can be reduced and dendritic failure of Li anodes can be forestalled to improve cycle lifetimes. Thus, it is critical to understand the effect of indentation or nanopatterning on the mechanical properties of Li metal, including the role of residual compressive stress. In this work, we have investigated the mechanical properties of Li metal by using an Atomic Force Microscope (AFM) to precisely control the indentation on the metal surface. Herein we demonstrate two different force regimes for performing mechanical indentation and scratching of the metal surface and determine the elastic modulus and hardness of Li metal. Using two different force regimes for performing mechanical indentation and scratching of the metal surface, we are able to determine the elastic modulus and hardness of Li metal.

## Results

**Figure 1:**
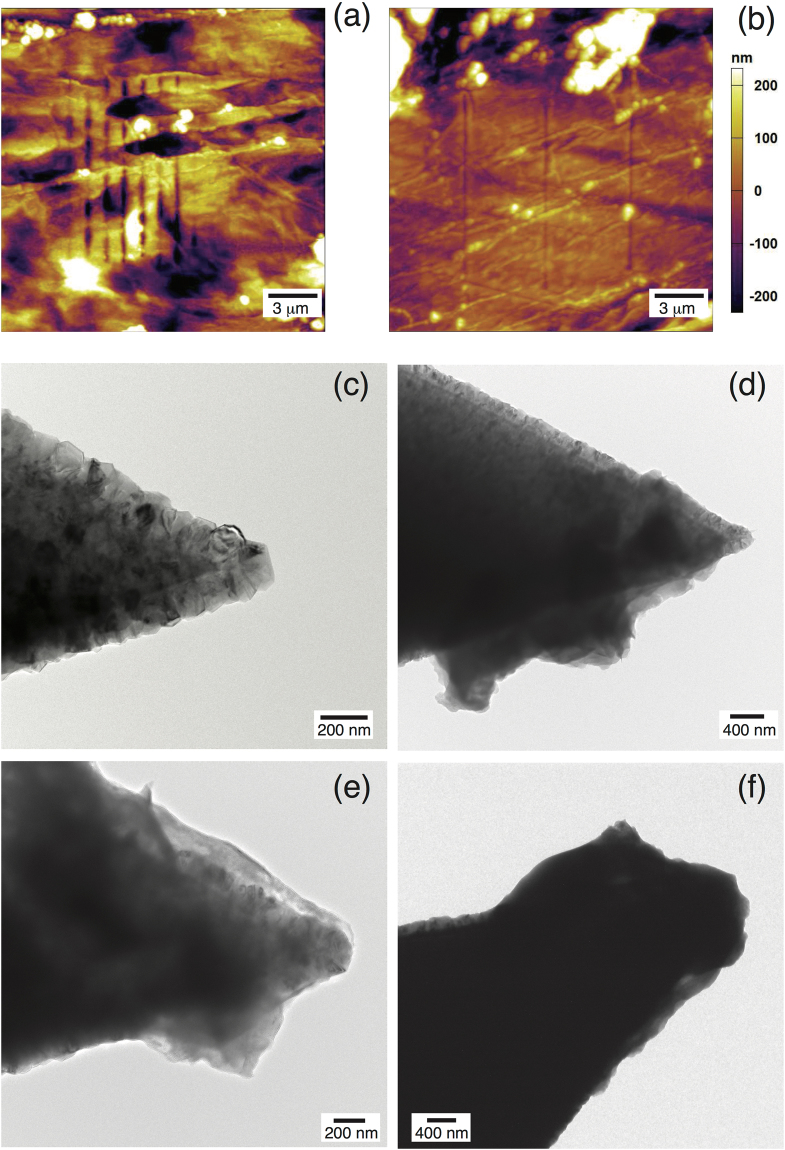
(**a**) Low-force scratching profile on Li metal. (**b**) High-force scratching profile on Li metal. (**c**) as received AFM tip before scratching experiments (**d**) AFM tip after successful low-force scratching. (**e**) AFM tip after one session of high-force scratching. (**f**) Subsequent image of the same tip in (**e**), after an additional high-force scratching session. Please note that the scale bar has changed, and that the tip is significantly damaged.

The experiments were performed on an as received Li metal foil sample (Sigma Aldrich, 99.9% trace metals basis). The mechanical scratching experiments were performed using a using silicon AFM tips with a hard, conductive, diamond-like-carbon coating. We used the high-force low-repetition scratching to estimate the hardness of the highly deformed lithium found at the edges of the scratch, and low-force high-repetition scratching to determine the hardness of unworked lithium. Based on the AFM images of high-force lithium scratching, we determined that ductile wear was significant (raised edges were observed on the sides of the sample, rather than sunken edges which could correspond to cracks). Additionally, *in-situ* force-distance (F/d) curves were taken and used to calculate the elastic modulus of Lithium under the assumption of Hertzian contact of a spherical indenter with the lithium^25^. TEM images of the AFM tips were taken before scratching to determine the radius of the tip accurately (average for the tips used: ~150 nm), and after scratching to determine the wear of the AFM tips. In Fig. 1, we illustrate the results of scratching experiments, and their effects on the tip morphology. These scratching experiments provide insight into the hardness of unworked Li, and that of highly deformed Li, both which will be discussed in the following sections.

**Figure 2:**
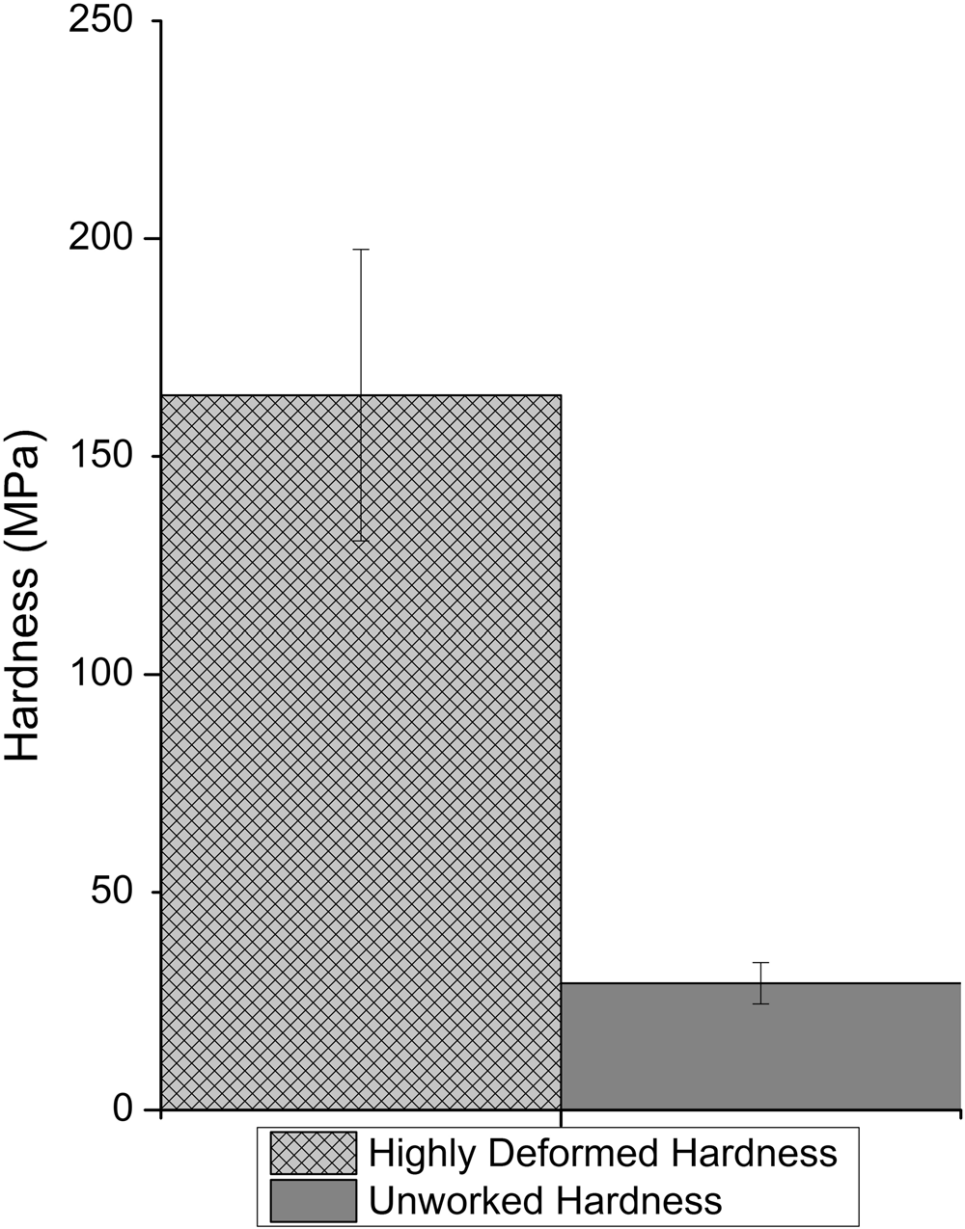
Hardness values measured/calculated. A significant difference is found between the highly deformed Li and unworked Li, so it is concluded that Li work hardens significantly.

 For the low-force scratching experiment, the data for the analysis of hardness of unworked lithium was measured from the region in which the depth of the scratch exceeded the approximate size of the spherical region of the tip. This was done to ensure that the scratch accommodates the tip completely, which was necessary to ensure that the area scratched by the tip was a known quantity: if  the scratched depth was too little, we would not have been able to accurately predict the hardness with the spherical tip approximation; if it was too high, we would not have measured the onset of plastic deformation. This methodology requires some small amount of plastic deformation, and so slightly overestimates the hardness of unworked Li. As can be seen in Fig. 1a), scratch depth can vary greatly based on the surface morphology (material was as-received from Sigma-Aldrich), so the largest persistent depth of scratch (maintained over the averaging of scan lines in the y-direction) was taken as the depth of the scratch, as even small surface features can cause significant hardening in the sample. The lowest force that could be said to have scratched the surface was divided by the contact area of the tip as calculated as a zone of a sphere using its approximate spherical radius and the depth of scratch formed. Using this method, we determined the unworked hardness of lithium as 29.1 ± 4.74 MPa, compared directly with the highly worked hardness in Fig. 2.

**Figure 3:**
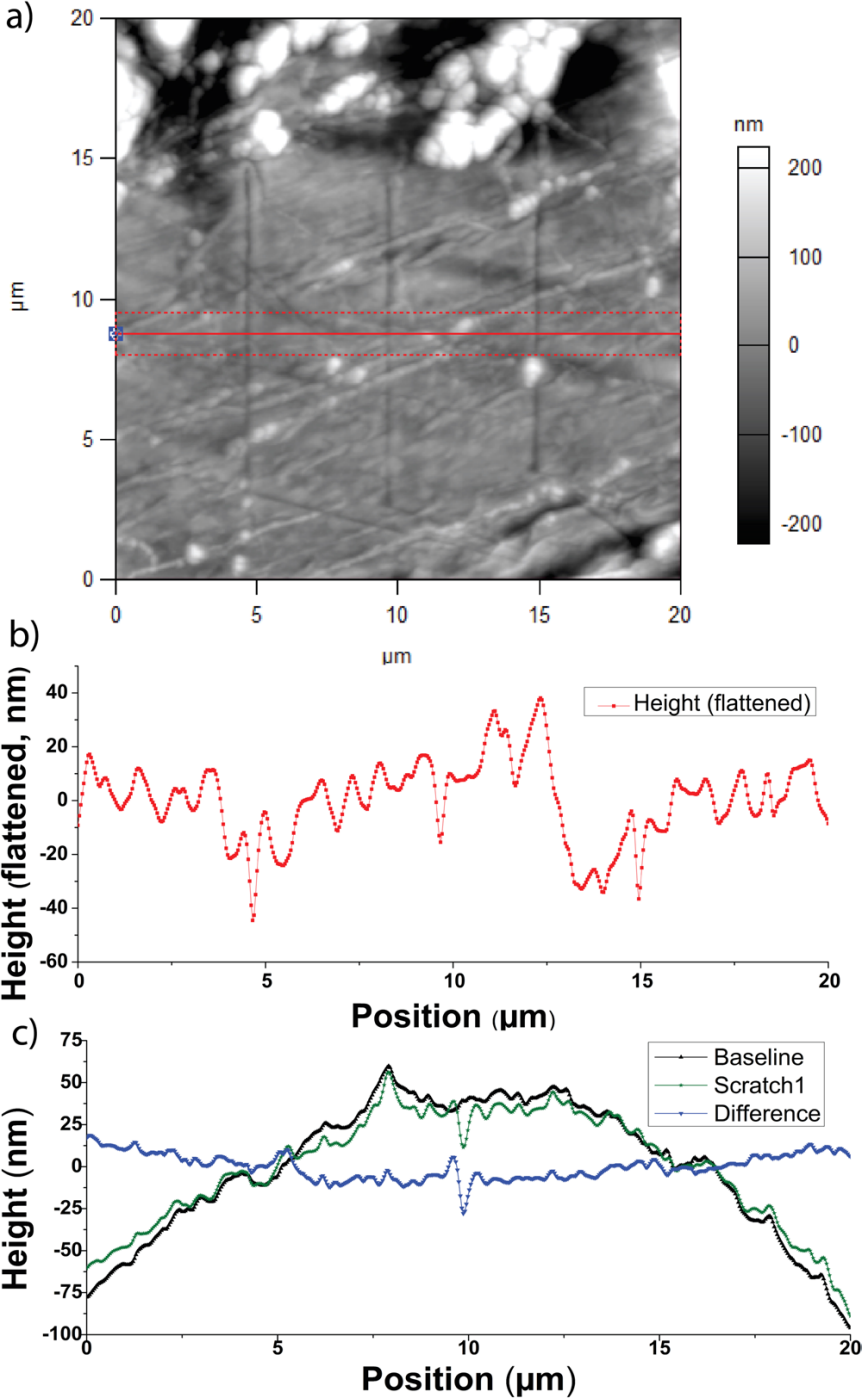
(**a**) Flattened scan of scratch area after 3 scratches. (**b**) flattened scratch profile, averaged over the region between the dotted red lines in (**a**). (**c**) Raw data and difference profile thereof, as is used to measure the scratch cross-section.

High-force single-pass scratching runs were performed to measure the hardness of highly deformed lithium. To calculate the hardness of Li in this manner, knowledge of the scratch cross-section is required. For this purpose, topographies were taken after every scratch and later compared to previous topographies, as illustrated in Fig. 3. It is important to note that finite tip size (“rolling ball”) corrections to these topographies have been made for the purposes of our calculations. Scratch cross-sectional topographies, such as the ones shown in Fig. 3, were acquired by comparing differences in AFM scans before and after the scratch was made in the area. A landmark was used to remove y-drift, and peaks in the cross-section were matched to remove x drift, minimizing the difference in cross-sections. In either case, the cross sections measured were the average height vs x-values of approximately 0.5 μm of y-values (30 scan lines). Scan angle was rotated so that scratches were imaged using a tip path orthogonal to the direction of the scratch. Using an equation derived from sections in “Microstructure and Wear of Materials”^26^, shown below (equation 1), the hardness of highly deformed Li was calculated to be 164 ± 33 MPa. A comparison of increase in hardness of highly deformed Li with unworked metal is shown in Fig. 2, showing approximately a fivefold increase in measured hardness. A more detailed derivation of this equation can be found in the supplementary information section.

1 $$\frac{{A}_{v}}{A}=\frac{{F}_{N}}{5A{H}_{def}}\sqrt{1+10{(\frac{{H}_{def}A}{K{F}_{N}})}^{2}}+\frac{{R}_{sector}^{2}}{A\,{\tan }\,\theta }(\frac{{(R/{R}_{{sector}})}^{2}({\theta }_{arc}-\,\sin \,{\theta }_{arc})\tan \,\theta }{2}-1)$$

### Determination of Elastic Modulus

The two prior methods calculate hardness, or more specifically the maximum pressure applied by the tip to the sample that does not yield plastic deformation. To determine how much elastic compliance a sample exhibits, the elastic modulus is required. This was calculated from force/distance curves under the assumption of Hertzian contact of a spherical indenter with an elastic half-space using the following relation:

2 $$\frac{F}{{d}^{3/2}}=\frac{4}{3}E\ast {R}^{1/2}$$

**Figure 4:**
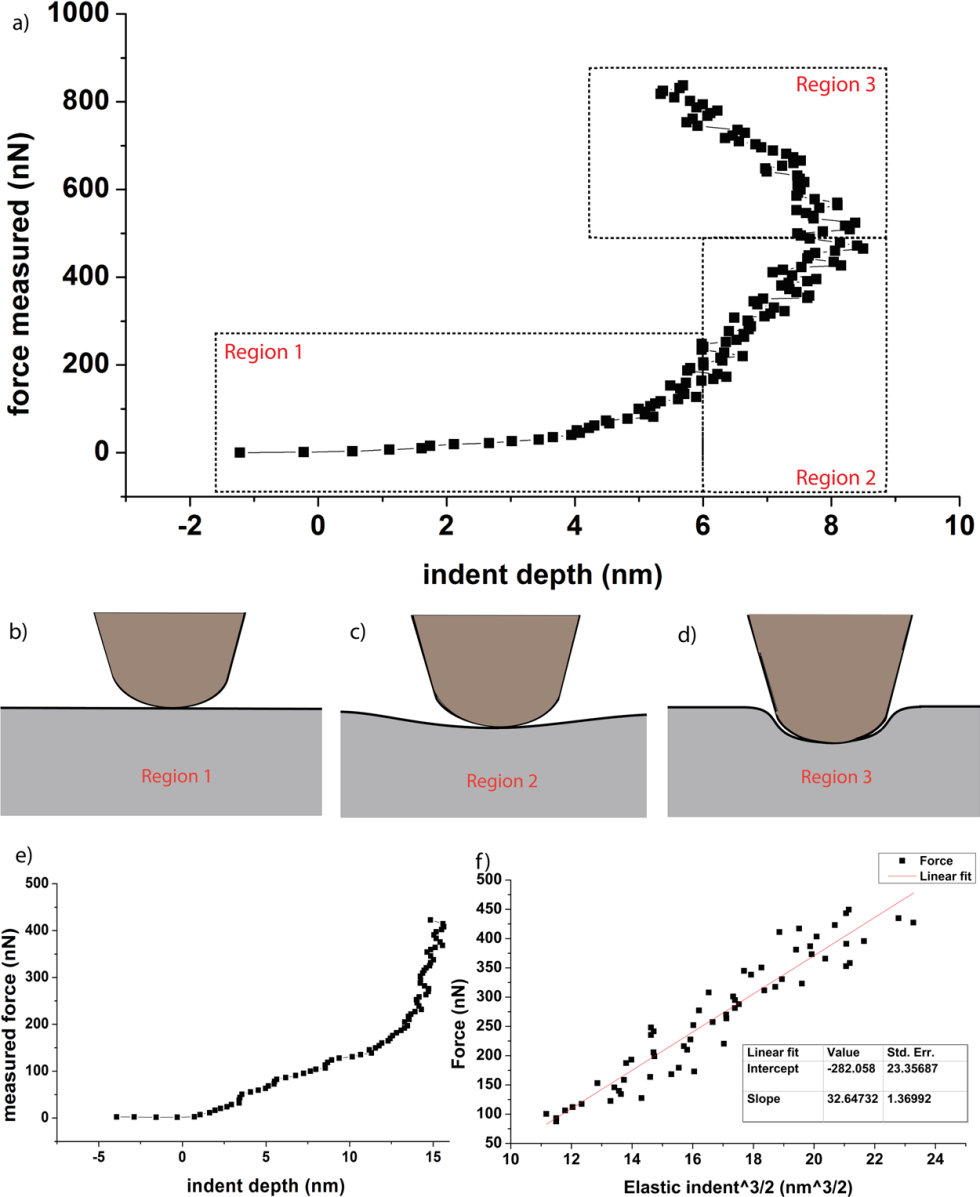
Illustration of the calculation of elastic modulus using the spherical indenter hertzian contact approximation in AFM. (**a**) is a Force vs. depth of indent curve used to calculate the elastic modulus, with peak force-applied greater than that required to produce plastic deformation. (**b**–**d**) illustrate schematically the different behaviors of the tip as the applied force increases. (**e**) is a Force vs. depth of indent curve for a maximum applied force less than that required to plastically deform the sample, and (**f**) is the data from region 2 of (**a**), plotted as force vs indent^3/2^, used to calculate the elastic modulus from the slope of a linear fit to the data.

F/d curves suggest reasonable agreement with this model (there are regions where the graph of F vs d^3/2^ is linear as shown in Fig. 4(f), and using the tip radius measured from TEM micrographs, an elastic modulus of 1.17 ± 0.55 GPa was calculated. There is one artifact we would like to note, which is visible in Fig. 4(a) and will be discussed in the next section.

## Discussion

While a significant variation in the mechanical properties of Li as a function of temperature has been reported elsewhere^27^, we do not believe frictional heating to be an issue in the low-force scratching measurements. Retraces of the tip’s path made during the scratching suggest that the majority of scratch formation occurs after several minutes of scanning at a rate of 1 Hz. If friction were a significant contributor to the results, deformation would be expected to have occurred after relatively few scratches due to the relatively high thermal conductivity of lithium (84.7 W/m-K)^28^. While the scan rate of the tip across the surface was decreased significantly for the high-force scratching runs, because so much material was deformed continuously the same argument cannot be made for high-force scratching. Fortunately, this works to improve the accuracy of our measurement: The highly-deformed hardness equation mentioned previously is based on an empirical model assuming a diamond indenter, and a front surface normal of this indenter that is parallel to the surface of the scratched sample^24^ (both of which are only approximate in our system). Because our tip is rounded significantly on the scale of the scratches made the hardness of highly worked lithium was somewhat overestimated, whereas heating from deformation would have softened the sample and offset these effects. That said, we attempted to minimize this effect by performing our single-pass high-force scratching experiments at approximately 0.1 Hz.

Also related to the high-force scratching measurements, the “rolling ball corrections” mentioned previously were only applied to the bottom of the scratch: the slope near the bottom of the scratch was evaluated, and the known effective radius of the tip was used to determine what width of the bottom of the scratch is lost due to a change in the section of the tip that is in contact with the sample. Other corrections, such as one that determines what depth is lost to the bottom of the sample by change in tip sampling region turn out to be exceedingly small. Because they are so small, and the tip is not completely spherical, these corrections have not been included.

In Fig. 1, debris is shown to coat the tip after scratching. This change in tip topography is unlikely to cause significant error in our measurements, as in the case of low force scratching, the debris is loosely adhered to the tip surface, as reflected by the burr-like profile in Fig. 1(d), as opposed to the tip-conforming one observed in Fig. 1(e). Other changes in tip topography are relevant though: While none are observed in the case of low-force scratching, Fig. 1(e) and (f) show the difference that even one session of high-force scratching makes. The radius measured after scratching was used for calculation of highly deformed lithium hardness from high-force scratching measurements.

Hardnesses calculated using these methods are in qualitative agreement with previous work by Xu *et al*.^26^. A yield stress (stress at the onset of plastic deformation, not that of failure) of 30–40 MPa at room temperature is reported in Xu’s paper, whereas we report one of 29 ± 5 MPa. While the ultimate yield strength reported in Xu’s work is significantly less than ours (105 MPa vs 164 ± 33 MPa), these should not be directly compared for two reasons: Firstly, the pillars in that investigation at room temperature failed by a single crystallographic shear mechanism that is not possible in a continuous medium, as is the case with lithium foil; and secondly, there is a non-trivial size difference between the sampled region in that paper and our work. In our work, the stress is distributed over a contact area of approximately 200 nm^2^, whereas that in Xu’s work distributes this stress over an area almost 25 times as large in its smallest limit.

Near zero indentation there is a relatively flat region (region 1, as defined in Fig. 4) where the Hertzian spherical indenter approximation is not valid, meaning the slope in this region is unfit for use in calculating the elastic modulus. At this point the contact area is extremely small, and because the contact force is so weak tip-interaction forces and friction are non-negligible, as are local non-spherical regions of the tip. As more force is applied to the tip, the contact area increases elastically (as in region 2, defined in Fig. 4) and the local non-spherical nature of the tip is of reduced importance. By determining the slope in this region, the elastic modulus may be calculated. The third region (region 3, in Fig. 4), displays a negative indentation distance which is caused by plastic deformation occurring beneath the tip. An AFM measures topological changes in constant-force contact mode by measuring the vertical position of the tip holder and the deflection of the tip. There is a ratio calculated at the beginning of every run called the Inverse Optical Lever Sensitivity (InvOLS), which is used to determine the distance deflected by a tip per unit change in the photodiode voltage used to measure its position. This ratio is calculated assuming an infinitely stiff sample. Plastic deformation in the material beneath a spherical indenter causes the yield stress of the material to increase: despite yielding, the material beneath the tip still exerts force on the tip. This in combination with a sudden increase in the load-bearing area that supports the tip causes a reduction in sample compliance (the amount by which the sample deforms per unit applied force), causing the tip to deflect substantially more than normal per unit change in tip holder height, resulting in the backwards swing seen in Fig. 4(a). As an example of the narrow margins in question here, the ~8 nm indentation achieved here occurred over a change in sensor head (and by extension, tip holder) height of about 150 nm, so even a small change in sample compliance is enough to cause significant error in the indentation depth, although the error in the elastic modulus calculated will only scale by factor change in the InvOLS. As further evidence of this analysis, see Fig. 4(e), a force vs indent curve taken immediately before that in 4(a): the maximum force applied in 4(e) does not exceed the force required to cause plastic deformation (which happens to be 550nN, near where the negative indent distance begins in 4(a), and a negative indentation distance is not observed. Where applicable, in curves that demonstrate this negative indentation distance behavior, the InvOLS has been reduced in our calculations until no such behavior occurred and a realistic value of the elastic modulus was calculated.

The value calculated by this method is 1.17 ± 0.55 GPa, with significant error likely arising from the incapability of differentiating crystallographic orientations independently of this measurement. This significantly differs from commonly reported literature values, such as those reported in Xu’s paper on the temperature and scale dependent mechanical properties of lithium metal^27^. Xu’s paper includes first-principles calculations which demonstrates reasonable agreement with previously collected data from a paper that used acoustic methods to determine elastic constants^29^. Both of these, especially the Projecter Augmented Wave (PAW) density functional theory calculations yield bulk quantities. The experiments in Xu’s paper similarly perform bulk sampling over pillars whose minimum height was 3 µm and minimum nominal diameter was 1 µm, whereas the AFM methodology reported in our paper distributes the vast majority of stress experienced by the sample in a region tens of nanometers in linear dimension; as such, it is possible our elastic modulus calculation represents a surface measurement, which should be noticeably different from bulk values. In addition to this, it is also possible that elastic modulus calculated in our paper is artificially low as a result of sampling issues: previous analysis demonstrates that stiff regions can be interpreted by the AFM as producing negative indentation distance, and can’t be used for elastic modulus calculation. If the elastic modulus of lithium in a region were to increase by a factor of ~10 as compared with the calibrating reference, as the data in Slotwinski’s paper suggests is possible for a change between <100> and <111> orientations^29^, this could be interpreted by the AFM as negative inflection distance. If that is the case, such a region would be indistinguishable from a highly concave region without further information, and as such may have been discarded in our analysis. This large anisotropy further suggests that using the polycrystalline Poisson’s ratio in the Hertzian contact calculation of elastic modulus is inappropriate, but varying that Poisson’s ratio between zero and 0.5 changes the calculated average by approximately 0.3 GPa, less than one standard deviation.

## Conclusions

We investigated the mechanical properties of patterned lithium metal surface using Atomic Force Microscopy. The hardness of unworked lithium was 29.1 ± 4.74 MPa which is much larger than the reported compressive strength of bulk Li (0.5 MPa), but size dependent properties have been reported previously so this sub-micron scale measurement’s deviation is not unexpected^24^. The elastic modulus was calculated from the force-distance curve to be 1.17 ± 0.55 GPa, and the hardness of highly deformed Li was calculated to be 164 ± 33 MPa. This suggests non-negligible work-hardening, and because both work hardening and residual stress are dependent on the dislocation density and stress field in the material it should be possible to generalize this finding to suggest that Lithium can bear a significant degree of residual stress. If Lithium does bear significant residual stress, that could inhibit the formation of new high-activity sites at the lithium anode during discharge and in doing so reduce the rate of dendrite formation and forestall dendritic failure. As such, we conclude that residual stress cannot be neglected as a factor affecting the performance of a lithium metal anode. In addition to a possible mechanism of improving cycle lifetime, this suggests that greater reproducibility of findings in other electrochemical experiments can be had by taking steps to guarantee a high-residual-stress or low-residual-stress state, and comparing like conditions.

## Methods

We prepared samples for AFM study of Li metal (Sigma Aldrich, 99.9% purity, trace metals basis) in an inert atmosphere environment (glove box atmosphere supplied with grade 5.0 Argon, maintained at <1.0 ppm H_2_O and O_2_) and subsequently transferred to the closed cell for AFM experiment (see supplementary information for further details). Due to the hazard of a loose sample colliding with the tip, a pair of small magnets were used to pin the lithium sample (approx. dimension 1 cm × 0.5 cm) to the glass bottom of the cell, and measurements were performed at the opposite end of the ~1 cm long sample. We operated the cell in a closed configuration, which prevents air from reaching the lithium sample for an extended duration, and after assembling the cell in the glove box, it was sealed and transferred to the AFM for mechanical indentation and scratching experiments. We scratched our samples using silicon AFM tips with a hard, conductive, diamond-like-carbon coating (Resonance frequency 65–155 kHz, Force Constant 1.5–18.3 N/m, 225 µm cantilever length, resistivity 0.01–0.02 Ωcm). After calibration using force-distance measurement (0.5–1 V relative deflection) and thermal spring constant determination, baseline topography images were taken in contact mode at approximately 0.5 V (<250 nN force applied) and 1 Hz over a 20 µm square area with the fast-scan direction perpendicular to the cantilever. Each AFM tip was used to scratch a line in the sample parallel to the cantilever in one of two ways: (i) High force, low repetition scratching in which the tip was dragged across the sample in one direction one time at high contact force (>5 µN) with .1 Hz scan rate over 10 µm scan length, and (ii) low force, high repetition scratching in which a small amount of force (<1 µN) was used to scratch the sample, repeatedly (512 times) in a section of the same area in both directions (10 or 15 µm scan length at same frequency as imaging, so lower scan speed than the baseline image). TEM micrographs were taken of the AFM tips before and after use to estimate the effect of wear during use on the tips as well as establish tip geometry.

## Electronic supplementary material

Supplementary Information
