# Supplementary material for: Effect of nanopatterning on mechanical properties of Lithium anode
Source: Sci Rep. 2018 Feb 6;8:2514. doi: 10.1038/s41598-018-20773-8 (PMC5802794; doi:10.1038/s41598-018-20773-8)
Supplement: Supplementary file 1 — Supplementary Information [file 41598_2018_20773_MOESM1_ESM.pdf]

Effect of nanopatterning on mechanical properties of Lithium anode

Colin Campbell,[a, b] Yong Min Lee,[c] Kuk Young Cho,[d] Young-Gi Lee,[e] Byeongdu Lee,[f] Charudatta Phatak,[a,*] and Seungbum Hong[g,1]

[a] *Materials Science Division, Argonne National Laboratory, Lemont, IL 60439, USA.*

[b] *Department of Materials Science and Engineering, Northwestern University, Evanston, IL 60208, USA.*

[c] *Department of Energy Science and Engineering, Daegu Gyeongbuk Institute of Science and Technology (DGIST), Daegu 42988, Korea.*

[d] *Deparment of Materials Science and Chemical Engineering, Hanyang University, Ansan 15588, Korea*

[e] *ICT Materials and Components Research Laboratory, ETRI, Korea.*

[f] *X-ray Sciences Division, Argonne National Laboratory, Lemont, IL 60439, USA.*

[g] *Department of Materials Science and Engineering, KAIST, Daejeon 34141, Korea.*

Supplementary Information

Illustration of closed fluid cell:

The electrical closed fluid cell used the experiments is shown in Figure S1. a) is the base of the closed fluid cell, note the three threaded holes used to seal the cell between the membrane and the brown O-ring shown here. In b) the membrane clamp assembly, showing how the membrane is connected to the cantilever holder for the AFM. This is the orientation in which the clamp can be placed atop the electrical closed cell and have its screws tightened into the base for an airtight seal. c) illustrates the membrane clamp assembly, showing how an AFM tip is loaded into the cantilever holder while the clamp is attached.

[1] Corresponding author: cd@anl.gov, seungbum@kaist.ac.kr

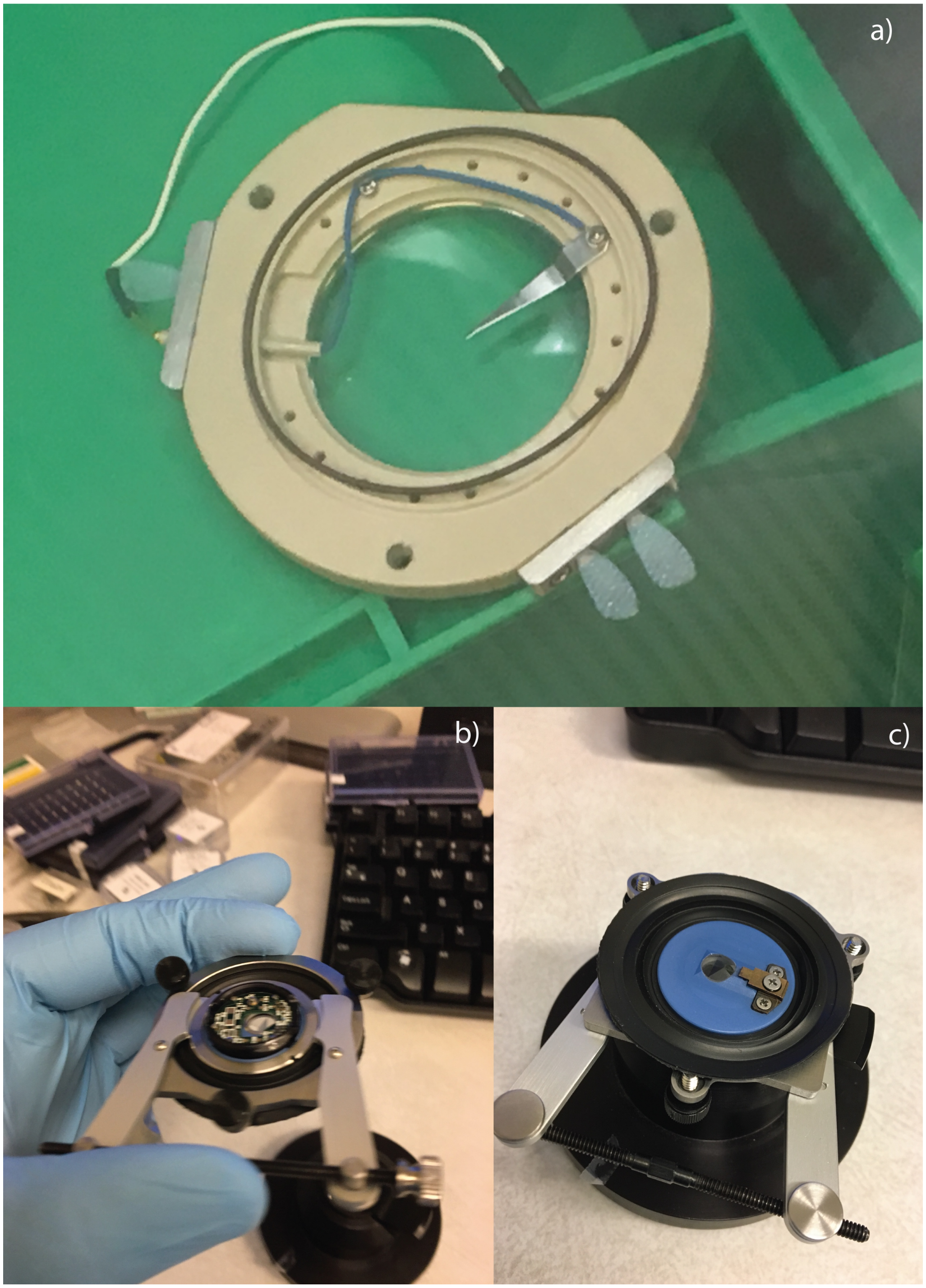

Figure S1: Images showing the closed fluid cell used in this work.

Derivation of High Force Scratching Equation:

Assuming material that is plastically deformed maintains its volume, f_{ab} expresses the ratio of the amount of material removed from the scratch to the amount of material deformed (Figure S2).[1]

$$f_{ab} = \frac{A_v - (A_1 + A_2)}{A_v} \tag{1}$$

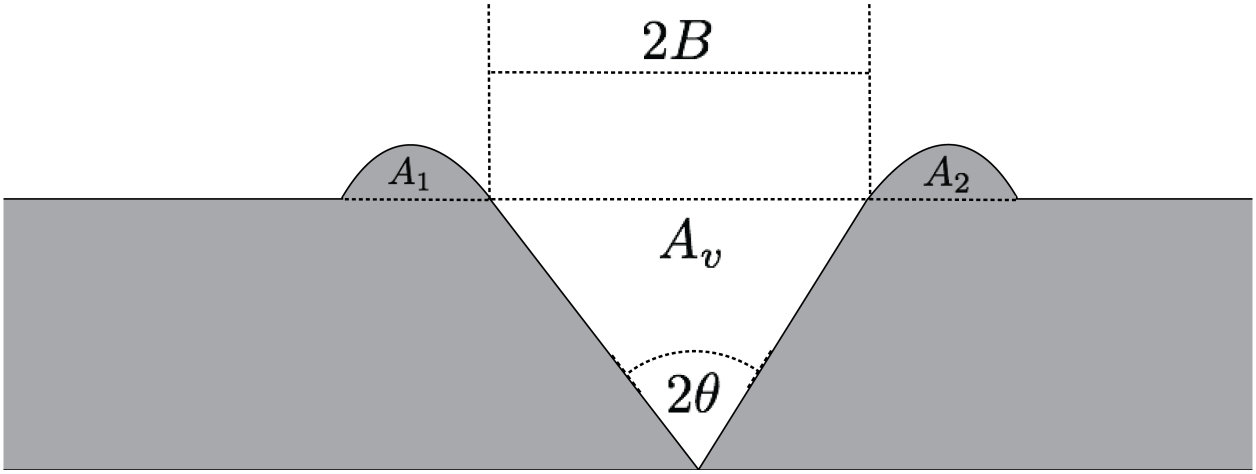

Figure S2: Schematic illustration of scratch profile and wear ratio.

Here A_v is the scratch area, and A_1, A_2 are the areas of the material raised on the edges of the scratch, B is the half-width of the scratch, and θ is the central half-angle of the scratch. Using the same definitions as above, the following must be true where $W_{v/s}$ is the volume of material removed per unit length of scratch:

$$W_{\frac{v}{s}} = f_{ab}A_v \tag{2}$$

The individual terms in above equations can be derived as follows. Assuming a form of the scratch profile to be trapezoidal, with a semicircular "cap", the scratch area, A_v can be calculated as follows.[1]

$$A_v = \frac{B^2 - R^2}{\tan(\theta)} + \frac{\pi R^2}{2} \tag{3}$$

Where R is an effective tip radius (radius of a rounded region at the bottom of the scratch trench, not shown in Figure S2). Using an approximation for the scratch width based on the assumption that a diamond is scratching the surface such that its forward face is exactly parallel to the surface normal perpendicular to the direction of the scratch[1]:

$$B^2 = \frac{F_N \tan(\theta)}{5H_{def}} \sqrt{1 + 10\mu^2} \quad (4)$$

Where H_{def} is the hardness of the material when highly deformed, and F_N is the force applied to the tip (normal force the sample exerts on the tip). This is expected to be approximately appropriate for our diamond-coated AFM tips. The coefficient of friction "μ" is taken to be the sum of two friction coefficients; one from adhesive friction and the other from the force required to push aside the amount of material from the scratch required to keep moving (the "grooving" coefficient of friction). Because the latter is expected to be much larger than the former for the high-force single-pass scratching, we approximate it as just the grooving friction term[1]:

$$\mu \approx \mu_g = \frac{\tau_c A}{F_N} \quad (5)$$

Here A is the area of contact between the tip and the sample, and τ_c is the shear strength of the material and can be related to the hardness of highly deformed material as[1]:

$$H_{def} \approx \tau_c K \, , \quad (6)$$

where K is the related to the number of slip systems in the material (>=5 required for plasticity). We chose $K = 24$, for our calculations based on the resolution of stress components along a single direction, and the 48 possible slip systems exhibited in BCC crystals. Substituting equation 6 into equation 5, we get,

$$\mu \approx \frac{H_{def} A}{K F_N} \quad (7)$$

Dividing the scratch area A_v by the area of contact of the tip A, and applying the previously defined grooving coefficient of friction and scratch width approximations, we get the following expression:

$$\frac{A_v}{A} = \frac{F_N}{5AH_{def}} \sqrt{1 + 10 \left(\frac{H_{def} A}{K F_N}\right)^2} + \frac{R^2}{A \tan(\theta)} \left(\frac{\pi \tan(\theta)}{2} - 1\right) \quad (8)$$

However, this expression assumes a hemispherical tip-end. In order to correct for that, we replace the "R" in the trapezoidal area term from the previous definition of A_v (equation 3) with the length of the chord of the tip's spherical (circular in profile) end whose height above the end of the tip is such that a continuous slope from tip-conformed scratch profile to trapezoidal scratch-profile is achieved, a length referred to here as R_{sector}; and replace the semicircular area term with the area between the arc of tip radius R and the chord whose length is $2R_{sector}$ and is parallel to the tangent of the bottom of the tip. An illustration of these quantities in a model cross section of a scratch is shown in the schematic Figure S3.

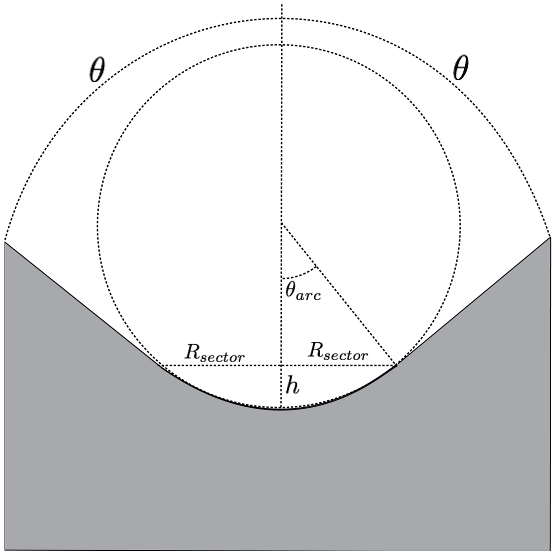

Figure S3: Schematic illustration of a model cross section of a scratch to correct for the hemispherical tip-end approximation.

The area between the chord R_{sector} drawn here and the edge of the tip-conformed bottom of the scratch is then given by:

$$A_{sector} = 2\left[\frac{R^2}{2}(\theta_{arc} - \sin(\theta_{arc})\cos(\theta_{arc}))\right] \quad (9)$$

Or, redefining θ_{arc} to be double the value used here (the total angle subtended by the non-trapezoidal scratch region, rather than the angle from the vertical), using the double angle formula for sines and substituting everything back into the hemispherical tip equation 8 with the corrections, we get the final equation as:

$$\frac{A_v}{A} = \frac{F_N}{5AH_{def}}\sqrt{1 + 10\left(\frac{H_{def}A}{KF_N}\right)^2} + \frac{R_{sector}^2}{A\tan(\theta)}\left(\frac{\left(\frac{R}{R_{sector}}\right)^2(\theta_{arc} - \sin(\theta_{arc}))\tan(\theta)}{2} - 1\right) \quad (10)$$

We can plot the wear area A_v in units of (A), as a function of the hardness in units of (F_N/A) using the above equation and the following parameters: $K = 24$, $\theta_{arc} = .0983\pi, \frac{R_{sector}^2}{A} = 0.06226, and \frac{R_{sector}}{R} = 0.153746$, and a variety of θ values shown in Figure S4.

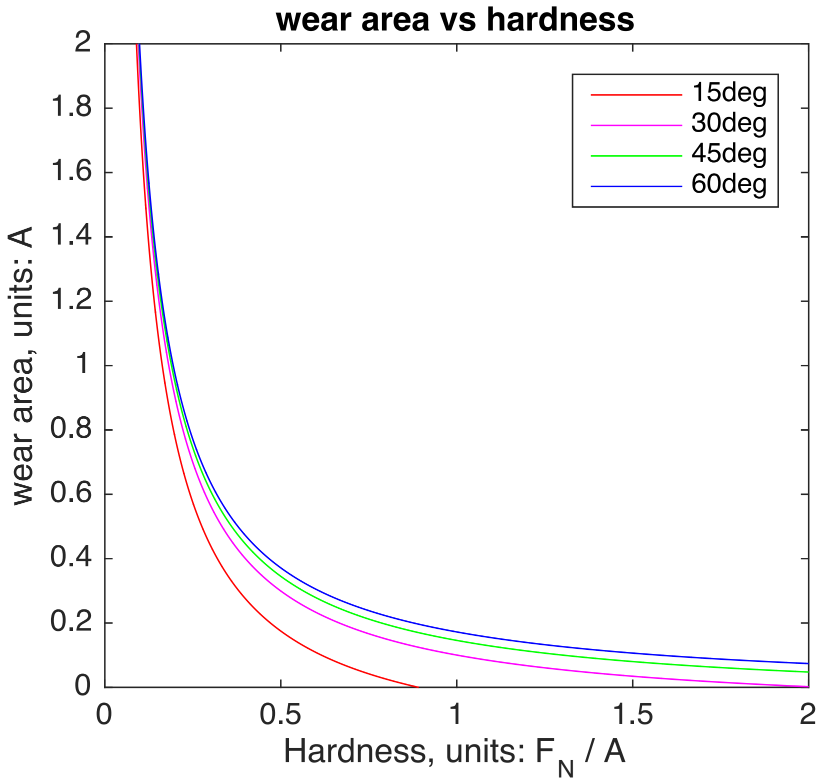

Figure S4: A graph showing the relation between wear area and hardness (equation 10) for various values of theta.

Reference:

[1] K. H. Zum-Gahr, *Microstructure and Wear of Materials*, 2nd Ed. (Elsevier, Amsterdam, NL, 1987).
